# 3D Printing of Hot Dog‐Like Biomaterials with Hierarchical Architecture and Distinct Bioactivity

**DOI:** 10.1002/advs.201901146

**Published:** 2019-08-08

**Authors:** Tian Li, Dong Zhai, Bing Ma, Jianmin Xue, Pengyu Zhao, Jiang Chang, Michael Gelinsky, Chengtie Wu

**Affiliations:** ^1^ State Key Laboratory of High Performance Ceramics and Superfine Microstructure Shanghai Institute of Ceramics Chinese Academy of Sciences No.1295 Dingxi Road Shanghai 200050 P. R. China; ^2^ Center of Materials Science and Optoelectronics Engineering University of Chinese Academy of Sciences No,19(A) Yuquan Road Beijing 100049 P. R. China; ^3^ Centre for Translational Bone Joint and Soft Tissue Research University Hospital Carl Gustav Carus and Faculty of Medicine of Technische Universität Dresden Fetscherstr. 74 01307 Dresden Germany

**Keywords:** 3D printing, hierarchical structure, hot dog‐like scaffolds, tissue regeneration

## Abstract

Hierarchical structure has exhibited an important influence in the fields of supercapacitors, catalytic applications, and tissue engineering. The hot dog, a popular food, is composed of bread and sausage with special structures. In this study, inspired by the structure of a hot dog, the strategy of combining direct ink writing 3D printing with bidirectional freezing is devised to prepare hot dog‐like scaffolds with hierarchical structure. The scaffolds are composed of hollow bioceramic tubes (mimicking the “bread” in hot dogs, pore size: ≈1 mm) embedded by bioceramic rods (mimicking the “sausage” in hot dogs, diameter: ≈500 µm) and the sausage‐like bioceramic rods possess uniformly aligned lamellar micropores (lamellar pore size: ≈30 µm). By mimicking the functions of hierarchical structure of bone tissues for transporting and storing nutrients, the prepared hot dog‐like scaffolds show excellent properties for loading and releasing drugs and proteins as well as for improving the delivery and differentiation of tissue cells. The in vivo study further demonstrates that both the hierarchical structure itself and the controlled drug delivery in hot dog‐like scaffolds significantly contribute to the improved bone‐forming bioactivity. This study suggests that the prepared hot dog‐like scaffolds are a promising biomaterial for drug delivery, tissue engineering, and regenerative medicine.

Hierarchical structure design is widely studied in various fields including supercapacitor,^1^ catalytic applications,^2^ and tissue engineering.^3^ In the past decades, it is found that the micro/nanoscale hierarchical structures have great influences on material properties.^4^ Thus, designing materials with specific micro–nanostructures has become a critical part in improving their diverse function and application. As an important multimaterials and multifunctional fabrication technology, direct ink writing (DIW) plays an important role in structure design.^5^ However, it is difficult to endow DIW 3D printing of materials with specific micro/nanostructure, due to the complexity and resolution limitation of printing nozzle and controlled system of printer instruments.^6^, ^7^

Large bone defect is still a thorny challenge in clinic because of the limited self‐repairing ability of bone tissues.^8^ Recently, implantation of 3D‐printed scaffolds is considered as an effective approach for stimulating bone regeneration.^9^ Human bones possess hierarchical structures from micrometer scale to nanometer scale,^7^, ^10^ which plays important roles in the transportation and storage of the nutrients and tissue cells.^11^ However, the conventional 3D‐printed scaffolds are composed of the stacked solid struts, but lack of hierarchical structures, limiting the delivery of nutrition and tissue cells, and further affecting the tissue formation in the inner of large bone defects.^12^

Hot dog, a common food constituted by bread and sausage which supply the energy and nutrition, respectively. In this study, inspired by the structure and function of hot dog, hot dog‐like scaffolds with hierarchical structure are successfully fabricated by combining DIW 3D printing and bidirectional freezing. The scaffolds comprise macroporous hollow tubes (bread) embedded by bioceramic rods (sausage) with uniformly aligned lamellar microstructures. The hot dog‐like structure could significantly enhance the specific surface areas of scaffolds and efficiently promote the cells adhesion inside; In addition, the sausage of hot dog can supply the nutrition source. Meantime, the interior hierarchical rods in this study can help the delivery of osteogenic drugs, which further contributes to the cell differentiation and bone formation. Therefore, it is reasonable to prepare hot dog‐like scaffolds from both structure and function points of view.

One of most interesting results of the study is that we successfully prepared hot dog‐like bioceramic scaffolds. **Figure** **1**a illustrated the typical fabrication process of hot dog‐like scaffolds by using the strategies of combined 3D printing and bidirectional freezing. Hollow tube bioceramic scaffolds were first prepared by 3D printing with modified nozzle (Figure S1a, Supporting Information). Then, the prepared scaffolds were put into the well‐dispersed bioceramic slurry for bidirectional freezing. During the bidirectional freezing process, lamellar ice crystals grew perpendicular to the steel plate due to the dual temperature gradients, while the bioceramic particles were squeezed to the position between two adjacent lamellar ice crystals (Figure S1b, Supporting Information). Subsequently, the ice crystals in the scaffolds were sublimated out through freeze‐drying, and the hot dog‐like scaffolds were finally achieved after being sintered.

**Figure 1:**
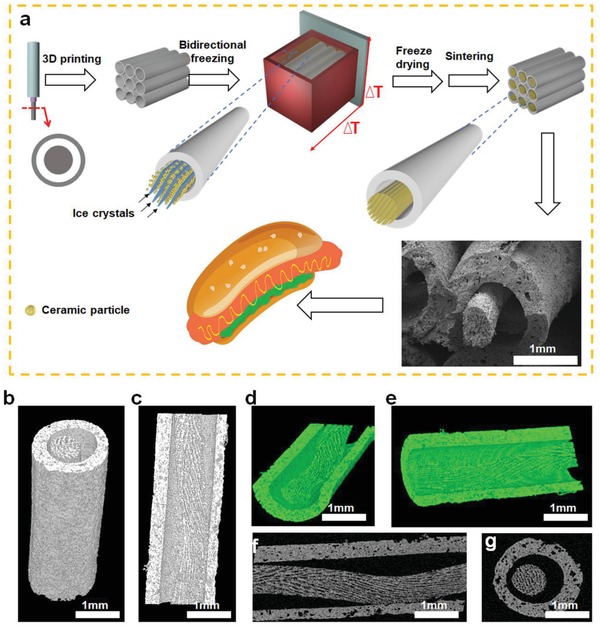
Fabrication and morphology of hot dog‐like scaffolds (HD‐AKT). a) The schemata of preparation of HD‐AKT, combining 3D printing and bidirectional freezing to realize 3D printed scaffolds containing rods with aligned lamellar microstructure. b–e) 3D micro‐CT images and f,g) 2D micro‐CT images of HD‐AKT from different views showing the scaffold structure, which is hollow tube macrospores embedded by bioceramic rods with the uniformly aligned lamellar structure of rods.

The hierarchical structure of hot dog‐like scaffolds was observed by the scanning electron microscopy (SEM). As shown in Figure 1a, the bioceramic rods with lamellar microstructure were embedded in the inner of the macroporous hollow tube of scaffolds. Micro computed tomography (Micro‐CT) images from different views showed that the structure of bioceramic rods inside the scaffolds was composed of large‐scale micro‐aligned lamellar framework (Figure 1b–g). Therefore, the hot dog‐like scaffolds possessed macroporous hollow tubes (bread) and microporous rods (sausage) with aligned lamellar framework.

Through traditional freeze‐casting, the microstructures and properties of materials could be controlled by regulating freezing temperature or slurry concentration.^13^ To well control the lamellar microstructure of the bioceramic rods in the scaffolds, we fabricated several kinds of scaffolds with different slurry concentration. In this study, akermanite (AKT, Ca_2_MgSi_2_O_7_), a representative silicate‐based bioceramic, was selected to fabricate the hot dog‐like scaffolds due to its excellent cytocompatibility.^14^

Hot dog‐like AKT(HD‐AKT) scaffolds with slurry concentrations: 20%, 30%, 40%, and 50% were fabricated and named as HD‐20AKT, HD‐30AKT, HD‐40AKT, and HD‐50AKT, respectively. To compare with hot dog‐like scaffolds, traditional solid struts AKT (S‐AKT) scaffolds and hollow tube AKT (H‐AKT) scaffolds without hot dog structure were fabricated for the controls. **Figure** **2**a showed the cross‐sectional morphologies of HD‐AKT scaffolds. The diameter of rods in HD‐AKT scaffolds increased with the increment of the concentrations of AKT slurry during the bidirectional freezing process. Meanwhile, the lamellar distance in the bioceramic rods decreased from 34 ± 1.0 to 18.5 ± 0.9 µm (Figure 2b), while the thickness of ceramic layers increased from 17.1 ± 1.3 to 66.8 ± 1.5 µm (Figure 2c). The porosities of the scaffolds obviously decreased from 43 ± 3.06% to 27.6 ± 1.32% with the increase of slurry concentrations (Figure 2d). By using the two‐step strategy, the hot dog‐like scaffolds with various materials including AKT (tubes)‐AKT (rods), Nagel (tubes)‐Nagel (rods), TCP (tubes)‐AKT (rods), and AKT (tubes)‐GO (rods) could be well prepared, suggesting the universality of the strategy (Figure 2e). Due to the advantage of asynchronous sintering, the sintering shrinkage of rods occurred in the second step, leaving the tube (bread) and rod (sausage) partly separated. It was observed from the micro‐CT images that some parts of the rod are sintered together with the inner of tube, showing the stability of the structure (Figure S2a, b, Supporting Information).

**Figure 2:**
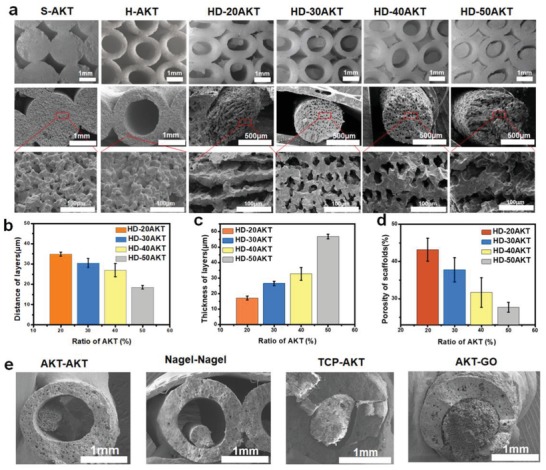
The hot dog‐like scaffolds are composed of hollow bioceramic tubes (mimicking the “bread” in hot dog) embedded by bioceramic rods (mimicking the “sausage” in hot dog) and the sausage‐like bioceramic rods possess uniformly aligned lamellar micropores. a) Morphology control of AKT scaffolds. Different structures of hot dog‐like scaffolds are obtained by changing the ratio of AKT in the freezing casting slurry from 20% to 50%, named for HD‐20AKT, HD‐30AKT, HD‐40AKT, and HD‐50AKT, respectively. Traditional 3D printed‐solid struts AKT scaffolds and 3D printed‐hollow tube scaffolds are named as S‐AKT and H‐AKT. The optical images (first line) and the SEM images show rods' lamellar structure of HD‐AKT. b) The characterizations of scaffolds: the distance of layers, c) the thickness of layers, and d) the scaffolds' porosity. e) Hot dog‐liked 3D printed scaffolds with different materials: AKT (tube)‐AKT (rods), Nagel (tube)‐Nagel (rods), TCP (tube)‐AKT (rods), and AKT (tube)‐GO (rods).

The second interesting result of this study is that the prepared hog dog‐like bioceramic scaffolds could be used for drug/protein delivery due to their hierarchical microstructure. Previous studies showed that the microstructure characteristics of biomaterials play critical roles for providing absorption sites of drug and protein, and further contribute to modulating their delivery.^15^ By mimicking the function of nutrition supply for sausages in hot dog, the potential of the scaffolds for loading icariin (Ica, a model osteogenic drug^16^), was investigated in this study. As shown in **Figure** **3**a,b, the loading efficiency and capacity of the Ica could be well controlled through changing the lamellar microstructure of bioceramic rods of HD‐AKT. HD‐30AKT and HD‐40AKT possessed the highest Ica loading efficiency up to 7.5% and 8 mg g^−1^ (mass ratio for drug/scaffold). However, the Ica loading efficiency and capacity of S‐AKT were much lower than that of HD‐AKT, indicating the excellent loading capacity of the hierarchical hot dog‐like scaffolds. The thermogravimetric analysis further verified the significantly improved loading efficiency of HD‐AKT scaffolds as compared to those without hot dog microstructure (Figure 3c).

**Figure 3:**
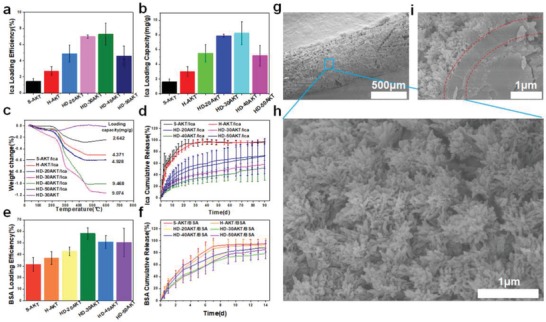
Hot dog‐like scaffolds are an excellent carrier for drug and protein. The drug Ica and protein BSA loading and release properties of the HD‐AKT. a) The Ica loading efficiency, and b) the loading capacity of traditional solid struts scaffolds(S‐AKT), H‐AKT, and different kinds of HD‐AKT. c) Thermogravimetric analysis of the scaffolds after loading the Ica. d) The Ica release of different kinds of scaffolds after loading Ica (S‐AKT/Ica, H‐AKT/Ica, HD‐20AKT/Ica, HD‐30AKT/Ica, HD‐40AKT/Ica, and HD‐50AKT/Ica). e) The BSA loading efficiency. f) The BSA release of the scaffolds. g–i) SEM images of the hot dog rod of scaffolds g) after 90 d Ica release. h) Surface images and i) the cross‐section image show lots of mineralized hierarchical structures on the surface of hot dog rod of scaffolds. Compared with the S‐AKT and H‐AKT, the HD‐AKT have higher loading efficiency and capacity. Meantime, HD‐AKT possess longer Ica/BSA release time.

Furthermore, the HD‐AKT scaffolds could maintain a sustained release of Ica for over 90 d, while Ica in S‐AKT and H‐AKT scaffolds completely released no more than 20 d, indicating that the hierarchical structure of HD‐AKT scaffolds plays a key role for maintaining the sustained release of drug. As compared with the S‐AKT and H‐AKT, the HD‐AKT scaffolds could provide more drug absorption sites due to the existence of lamellar structures which increase the specific surface area of HD‐AKT scaffolds (Table S1, Supporting Information). In addition, it was found that plenty of mineralized calcium phosphate microcrystals formed on the surface of the rods, which could contribute to the slow release of the Ica from scaffolds (Figure 3g–i and Figure S4, Supporting Information).^17^

Not only the small molecule drug Ica but also the large molecule protein bull serum albumin (BSA) could be well delivered by HD‐AKT scaffolds. Similar with Ica loading, HD‐AKT exhibited higher loading efficiency of BSA than H‐AKT and S‐AKT (Figure 3e). In addition, HD‐AKT scaffolds displayed longer period of BSA release time than other scaffolds (Figure 3f).

The third interesting result of the study is that the prepared hot‐dog like scaffolds possess excellent bioactivity both in vitro and in vivo. To explore the effect of hot dog structure of HD‐AKT scaffolds on the cell delivery and osteogenic differentiation of rabbit bone mesenchymal stem cells (rBMSCs), the cells were cultured on the S‐AKT, H‐AKT, HD‐30AKT, HD‐30AKT/Ica, respectively. As shown in **Figure** **4**a–d, rBMSCs were well attached to the scaffolds after 3 d of culture. The rBMSCs could be delivered into the inside of the hierarchical struts of HD‐30AKT and HD‐30AKT/Ica; however, the cells were only attached on the outside surface of solid struts of S‐AKT (Figure 4a_2_–d_2_). Interestingly, there were lots of cells attached on the surface of lamellar rods in the HD‐30AKT from longitudinal section view (Figure 4e). Cell proliferation assays revealed that rBMSCs in HD‐AKT and HD‐30AKT/Ica scaffolds with hot dog structure exhibited better activity than those in S‐AKT and H‐AKT indicating that the hierarchical structures of hot dog‐like scaffolds could be beneficial for the proliferation of rBMSCs, due to the higher specific surface area of lamellar rods which surfaces were conductive to the adhesion of cells (Figure 4f). Moreover, HD‐30AKT and HD‐30AKT/Ica significantly enhanced the bone‐related gene expressions of rBMSCs (Figure 4g–j). Figure 4g showed that HD‐30AKT scaffolds significantly promoted the expression of runt‐related transcription factor 2 (Runx2) compared with H‐AKT scaffolds, which might be explained that more cells delivered on the rods might accelerate the gap junctions and regulated the Runx2 furthermore.^18^ Due to the effective release of Ica from the lamellar rods in the scaffolds, bone‐related gene expressions osteocalcin (OCN), osteopontin (OPN), alkaline phosphatase (ALP) were essentially increased as compared to other groups without Ica delivery (Figure 4h–j), suggesting that the prepared scaffolds are quite useful platform for drug delivery with significantly improved cell function toward to osteogenic differentiation for bone tissue regeneration.

**Figure 4:**
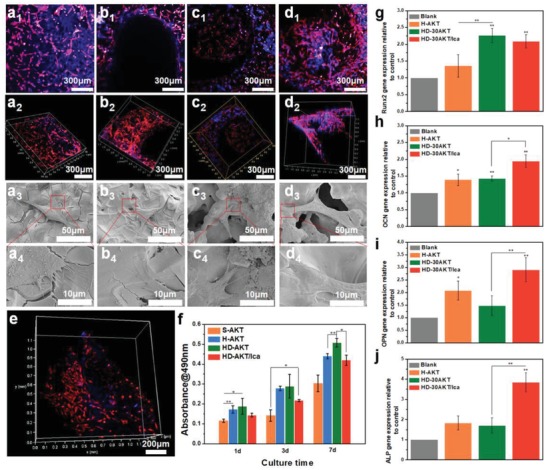
Hot dog‐like scaffolds are an excellent platform for cell delivery and differentiation. The proliferation, morphology, and relative genes expression of rBMSCs cultured on different scaffolds. a‐d) Confocal and SEM images of rBMSCs cultured on a) S‐AKT, b) H‐AKT, c) HD‐30AKT, and d) HD‐30AKT/Ica. e) The rBMSCs (red color) adhesion on the rod of HD‐AKT. f) The proliferation of rBMSCs after seeding in different kinds of scaffolds, showing hot dog‐like scaffolds are beneficial for cell proliferation. g) The relative osteogenic genes expression (OCN, Runx2, OPN, and ALP) of rBMSCs in scaffolds, indicating that the Ica release from the scaffolds promotes the relative osteogenic genes expression of rBMSCs (*n* = 6, *P < 0.05, and **P < 0.01.).

To further investigate in vivo bone‐forming bioactivity, S‐AKT, H‐AKT, HD‐30AKT, and HD‐30AKT/Ica were implanted into rabbit femoral defect for 8 weeks. Then, the overall photographs and micro‐CT analysis of femoral defect samples were displayed in **Figure** **5**a. The results demonstrated no inflammatory reaction in the defects (Figure 5a_1_–d_1_). Meantime, the newly formed bone tissues had grown into the scaffold tubes of H‐AKT, HD‐AKT, and HD‐AKT/Ica from the micro‐CT images of transverse view as indicated in Figure 5a_2_–d_2_ and a_3_–d_3_. The sagittal view of micro‐CT showed that only the parts near the tissue had formed new bones for H‐AKT (Figure 5b_4_). However, considerable amount new bones were formed in the induction of HD‐AKT and HD‐AKT/Ica scaffolds (Figure 5c_4_,d_4_). Micro‐CT reconstruction analysis showed that HD‐30AKT and HD‐30AKT/Ica scaffolds had higher the volume ratio of the new bone to the original defect regions (BV/TV) as compared to Blank, indicating superior bone repair ability of HD‐30AKT and HD‐30AKT/Ica (Figure 5e). Interestingly, HD‐AKT/Ica were conductive to better repair bone defects as compared with HD‐AKT, verifying that the hierarchical hot dog‐like scaffolds with Ica delivery effectively improve bone‐forming bioactivity. As exhibited in Figure 5g of bone staining images, there were few new bones in the Control group without the scaffolds. However, plenty of new bones (red) emerged and grew along the materials with the inducement of scaffolds (black) in Figure 5h–j. In addition, bone tissues were found near the rods of HD‐AKT scaffolds (blue circle) in Figure 5i, j compared with H‐AKT of Figure 5h. The results illustrated that the hot dog‐like scaffolds could significantly promote the osteogenesis by inducing the new bone to grow into the hierarchical structure rods of the scaffolds. Moreover, HD‐AKT/Ica (Figure 5j) exhibited more new bone than HD‐AKT (Figure 5i). Furthermore, the newly formed bones were observed to grow in the interior of the lamellar microstructures of rod (green arrows) from the stained images (Figure 5f), due to more nutrition and cell absorption sites provided from the hierarchical rods. Therefore, both hierarchical structure and the Ica delivery of the hot dog‐like scaffolds play a significant role for the regeneration of bone defects.

**Figure 5:**
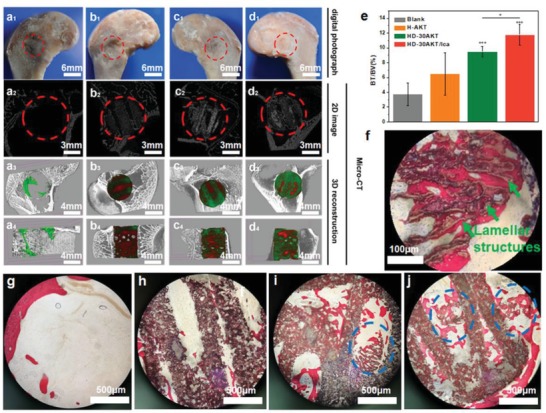
Hot dog‐like scaffolds possess excellent bone‐forming bioactivity after implanted in the femoral defects of rabbits. The characterizations of hot dog‐like scaffolds for osteogenesis in vivo. a_1_–d_1_) Digital, a_2_–d_2_) 2D micro‐CT, and 3D micro‐CT images (a_3_–d_3_: transverse view, and a_4_–d_4_: sagittal view) of the defects at week 8. In 3D micro‐CT images, green, red and white represents new bone, scaffold, and primary bone, respectively. e) Micro‐CT reconstruction analysis exhibits the volume ratio of the new bone to the original defect regions (BV/TV) at week 8. HD‐30AKT and HD‐30AKT/Ica indicate significantly. f) The newly formed bones (red) grow into the lamellar microstructures (green arrows) of hot dog‐like scaffold. g–j) Hard histological sections stained with Van Gieson's picrofuchsin of g) Blank, h) H‐AKT, i) HD‐AKT, and j) HD‐AKT/Ica, red color stands newly formed bone and black color represents scaffolds. Newly formed bone can grow into the hierarchical rods of the scaffolds (blue circle point to the new bone in the hierarchical rods). improvement in new bone regeneration as compared to Blank control. In addition, Ica can also promote osteogenesis, suggesting that both hierarchical structure and the Ica delivery of the hot dog‐like scaffolds contribute to the bone regeneration (*n* = 6, *P < 0.05, **P < 0.01, and ***P < 0.001.).

In summary, inspired by the constitution, structure and function of hot dog, we successfully fabricated the hierarchical hot dog‐like scaffolds which consisted of hollow tube embedded by bioceramic rods with uniformly aligned lamellar microstructures by combining DIW 3D printing with bidirectional freezing. The prepared hot dog‐like scaffolds exhibited hierarchical microstructure with improved specific surface area, which significantly enhanced the drug/protein delivery, cell proliferation, and osteogenic differentiation of rBMSCs. Due to the hierarchical lamellar microstructures and Ica delivery in scaffolds, the hot dog‐like scaffolds could significantly promote the formation of new bone tissue. Our study suggests that the hot dog‐like scaffolds can be used for the multifunctional biomaterials for drug delivery, tissue engineering, and regenerative medicine. The combined strategy of DIW 3D printing with bidirectional freezing is a promising method to prepare biomimetic and hierarchical biomaterials.

## Experimental Section

*Preparation of Hollow Tube Scaffolds by 3D Printing*: The printing ink was prepared by mingling 2.0 wt% sodium alginate (Alfa Aesar), 20 wt% Pluronic F‐127 (Sigma–Aldrich) polymer solution, bioceramic powder AKT (Ca_2_MgSi_2_O_7_), which was synthesized according to our previous publication.^19^ Modified printing nozzle was designed with a needle embedded in the nozzle (Figure S1a, Supporting Information) to prepare the hollow tube scaffolds by 3D printing based on our previous work.^20^ After drying in the room temperature overnight, the hollow tube AKT scaffolds were sintered at 1350 °C for 3 h. The traditional solid struts AKT scaffolds without hollow tubes were printed by conventional nozzle as the control group. Other bioceramic powders with different composition (e.g., β‐TCP, Nagel) were synthesized by the published methods.^21^

*Preparation of Hot Dog‐Like Scaffolds*: Hot dog‐like scaffolds were successfully fabricated by ice crystals growth into the hollow tube scaffolds in the bidirectional temperature gradients. The slurry was first prepared through mixing the AKT powders (20, 30, 40, and 50 vol%) with 2 wt% of polyvinyl alcohol, assisting 1 wt% of sodium polyacrylate as the dispersant, and then put into the vacuum oven for 15 min to remove the gas. Subsequently, the hollow tube scaffolds were placed into the mold in the direction of vertical to the steel as shown in (Figure S1b, Supporting Information) before adding the slurry into the mold. Dual temperature gradients, along and perpendicular to the steel plate, could be generated after the liquid nitrogen was poured into the box, so that the ice crystals would grow into the tubes. Further, the samples were freezing dried for 48 h to sublimate out the ice crystals and then the outside of the scaffolds can be removed easily. Ultimately, the hot dog‐like scaffolds were obtained after sintered at 1350 °C for 3 h.

*Characterization of the Hot Dog‐Like Scaffolds*: The scaffolds macroscopic morphologies were taken by optical microscopy (S6D, Leica, Germany). The microstructures were observed by micro‐CT (SKYSCAN1172, SKYSCAN, Belgium) and SEM (JSM‐6700F, Japan). The thickness of the layers and the distance between the layers were manually measured on basis of the SEM images. Ten measurements were performed at least for each parameter. The porosity was measured through Archimede method. In brief, the scaffolds were dried at 120 °C for 10 h first, weighed, and marked as M_1_. After that, the scaffolds were immersed in water and placed under vacuum for more than 2 min until no bubbles came up. The scaffolds with water‐filled pores were weighed and marked as M_2_. Finally, the buoyant weight of scaffolds was marked as M_3_. The porosity (P) was calculated by following formula (1).

(1) $$P\;\;=\;\;\left(M_{2}\;\;-\;\;M_{1}\right)\text{/}\left(M_{2}\;\;-\;\;M_{3}\right)\;\;\times \;\;100\%$$

*Characterization on the Ica and BSA Loading and Release of the Hot Dog‐Like Scaffolds*: For the Ica and BSA loading, the Ica absorption wavelength of 365 nm was acquired with UV/vis (Figure S2a, Supporting Information). In addition, the absorption standard curve was measured through Microplate Reader (Epoch, BIO‐TEK, USA, Figure S2b, Supporting Information). The scaffolds were soaked in Ica solution (50 mg mL^−1^) and BSA solution (20 mg mL^−1^) respectively for 3 d at 37 °C. Then the scaffolds were taken out and concentrations of the remaining solution were measured. To further test the amount of the release of Ica and BSA in the scaffolds, the samples were soaked in phosphate buffered saline (PBS) again and the medium was collected for concentration analysis using microplate reader and replaced with fresh PBS solution immediately at certain time interval. The loading efficiency, loading capability and released ability of Ica and BSA were calculated as following Equations (2) and (3).

(2) $$\text{Loading}\;\text{efficiency}\left(\%\right)\;\;=\;\;\left(C_{0}\;\;-\;\;C_{1}\right)\text{/}C_{0}\;\;\times \;\;100\%$$

(3) $$\text{Loading}\;\text{capability}\left(\%\right)\;\;=\;\;m_{1}\text{/}m_{2}\;\;\times \;\;100\%$$

where *C*_0_ is the concentration of Ica and BSA before loading and *C*_1_ refers to the concentration of Ica and BSA after loading, and *m*_1_ is the loading mass of Ica and BSA, in addition, *m*_2_ refers to the mass of the scaffolds.

*In Vitro Bioactivity of the Hot Dog‐Like Scaffolds*: The rabbit bone marrow stem cells (rBMSCs) were cultured in Dulbecco's Modified Eagle's Medium (DMEM, HyClone, China) supplemented with 10% fetal bovine serum (Invitrogen), penicillin‐streptomycin solution (Invitrogen). For the research of cell adhesion, rBMSCs were seeded on the scaffolds with the cell amount of 3 × 10^4^ in 48‐well culture plates. After incubation for 3 d, cells were fixed on the scaffolds by using 2.5% glutaraldehyde. Cell dehydration was accomplished through soaking in graded ethanol (30, 50, 70, 90, 95, and 100 v/v%). Before SEM analysis, the samples were dried by Hexamethyldisilazane. For better observation of the cell distribution, cells were fixed with 4% paraformaldehyde solution and stained with 4,6‐diamino‐2‐phenyl indole (DAPI) and rhodamine phalloidin. Then, confocal images were obtained through fluorescence confocal microscopy (TCS SP8, Leica, Germany). To evaluate cell proliferation, the rBMSCs were cultured on different scaffolds for 1, 3, and 7 d for MTT assay at 490 nm by a microplate reader. To explore the expression of bone relative gene, the rBMSCs were cultured in differentiation medium (DMEM supplemented with 10 × 10^−3^ M β‐glycerol phosphate, 0.2 × 10^−3^ M ascorbic acid, and 10% fetal bovine serum). After incubation for 7 d, the RNA was extracted using Trizol Reagent (Invitrogen Pty Ltd, Australia). The expression of relative bone genes was measured by RT‐qPCR: Runx2, OCN, OPN, and ALP.^22^

*In Vivo Bioactivity of the Hot Dog‐Like Scaffolds*: All the animal experiments were carried out in compliance with the relevant laws. And all procedures were approved by The Ethics Committee of Nanjing First Hospital, Nanjing Medical University. 12 New Zealand white rabbits (2.5–3 kg) were selected to evaluate the osteogenesis processes through implanting three kinds of scaffolds (H‐AKT, HD‐30AKT, and HD‐30AKT) into the critical‐sized femoral defect (diameter: 6 mm, and height: 8 mm), and the samples without the scaffold (Blank) were used as the control. After implanting for 8 weeks, the rabbits were sacrificed and the samples were gleaned. To estimate the osteogenesis, the samples were observed by micro‐CT (SKYSCAN1172, andSKYSCAN). Furthermore, histological images were acquired to evaluate the newly formed bone tissues after dehydrated, embedded in PMMA, sliced, and Van Gieson's picrofuchsin stained.

## Conflict of Interest

The authors declare no conflict of interest.

## Supporting information

Supplementary
