# Supplementary material for: 3D Printing of Hot Dog‐Like Biomaterials with Hierarchical Architecture and Distinct Bioactivity
Source: Adv Sci (Weinh). 2019 Aug 8;6(19):1901146. doi: 10.1002/advs.201901146 (PMC6774059; doi:10.1002/advs.201901146)
Supplement: Supplementary file 1 — Supplementary [file ADVS-6-1901146-s001.pdf]

Copyright WILEY-VCH Verlag GmbH & Co. KGaA, 69469 Weinheim, Germany, 2019.

Supporting Information

for *Adv. Sci.,* DOI: 10.1002/advs.201901146

3D Printing of Hot Dog-Like Biomaterials with Hierarchical Architecture and Distinct Bioactivity

*Tian Li, Dong Zhai, Bing Ma, Jianmin Xue, Pengyu Zhao, Jiang Chang, Michael Gelinsky, and Chengtie Wu**

Supporting Information

3D Printing of Hot Dog-like Biomaterials with Hierarchical Architecture and Distinct Bioactivity

*Tian Li, Dong Zhai, Bing Ma, Jianmin Xue, Pengyu Zhao, Jiang Chang, Michael Gelinsky and Chengtie Wu**

Table S1. The specific surface area of S-AKT, H-AKT, HD-20AKT, HD-30AKT, HD-40AKT, HD-50AKT.

Groups	Specific surface area ($\times 10^{-3} m^2/g$)
S-AKT	1.20 ± 0.12
H-AKT	1.68 ± 0.08
HD-20AKT	4.33 ± 0.10
HD-30AKT	9.88 ± 0.11
HD-40AKT	7.99 ± 0.22
HD-50AKT	5.09 ± 0.17

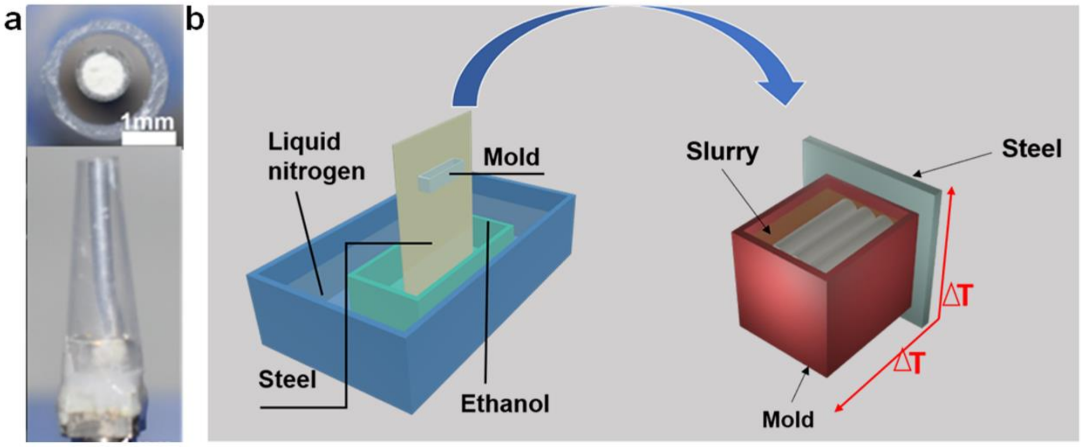

Figure S1. a) The optical images of modified nozzle. b) Bidirectional freezing device and the freezing process.

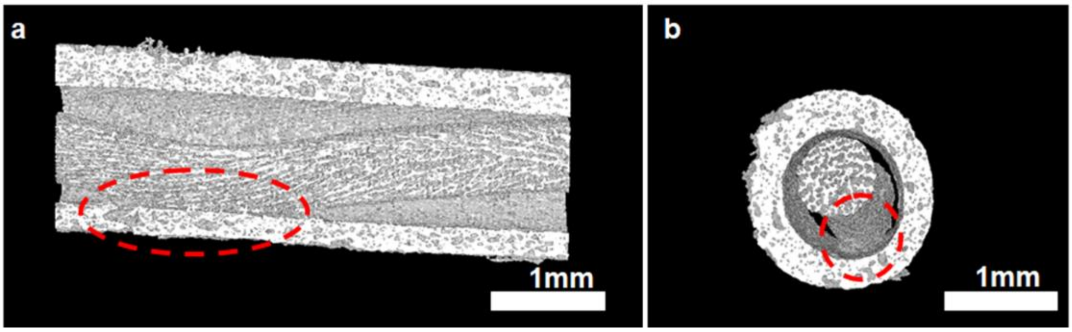

Figure S2. Micro-CT images of hot dog-like scaffolds a) Longitudinal section view b) cross- section view. The red circles refer to the connection parts of tube and rod.

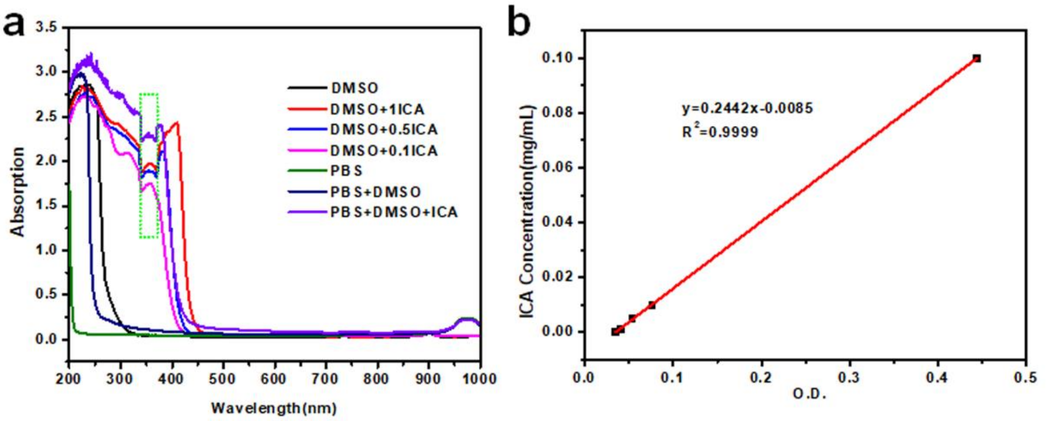

Figure S3. a) UV-vis indicating that the ICA has an absorption at 365nm. b) The ICA standard absorption curve measured at 365nm by Microplate Reader.

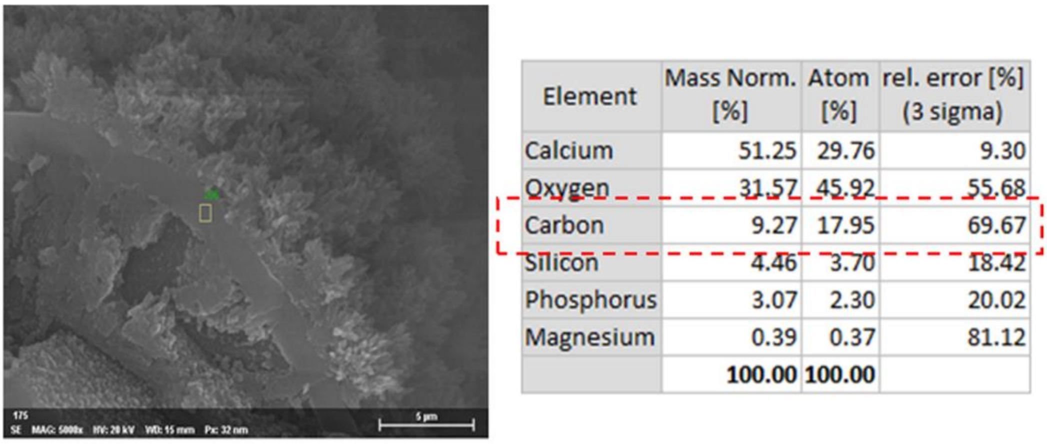

Element	Mass Norm. [%]	Atom [%]	rel. error [%] (3 sigma)
Calcium	51.25	29.76	9.30
Oxygen	31.57	45.92	55.68
Carbon	9.27	17.95	69.67
Silicon	4.46	3.70	18.42
Phosphorus	3.07	2.30	20.02
Magnesium	0.39	0.37	81.12
	**100.00**	**100.00**	

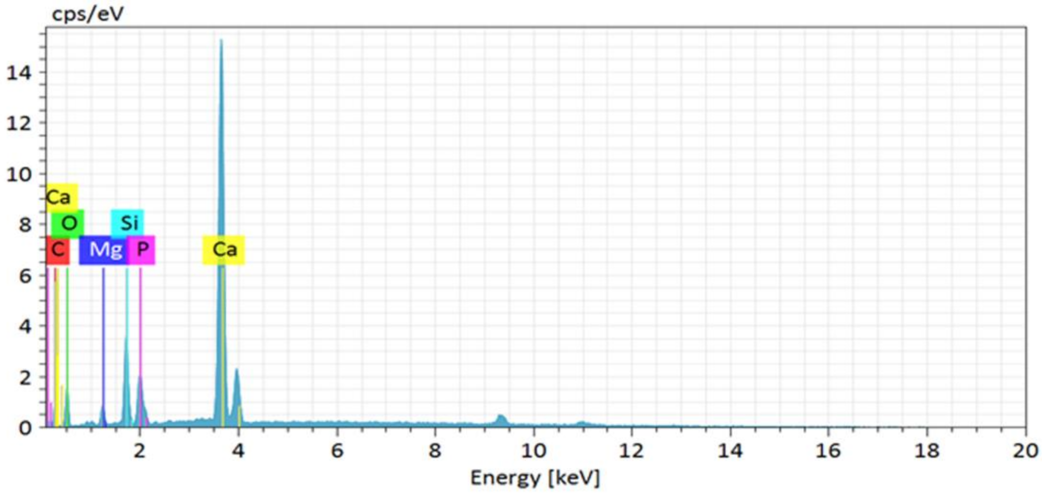

Figure S4. EDS analysis of the mineralization layer on the surface of the rods of hot dog-like scaffolds after release of drugs in PBS. The carbon comes from the unreleased drug.

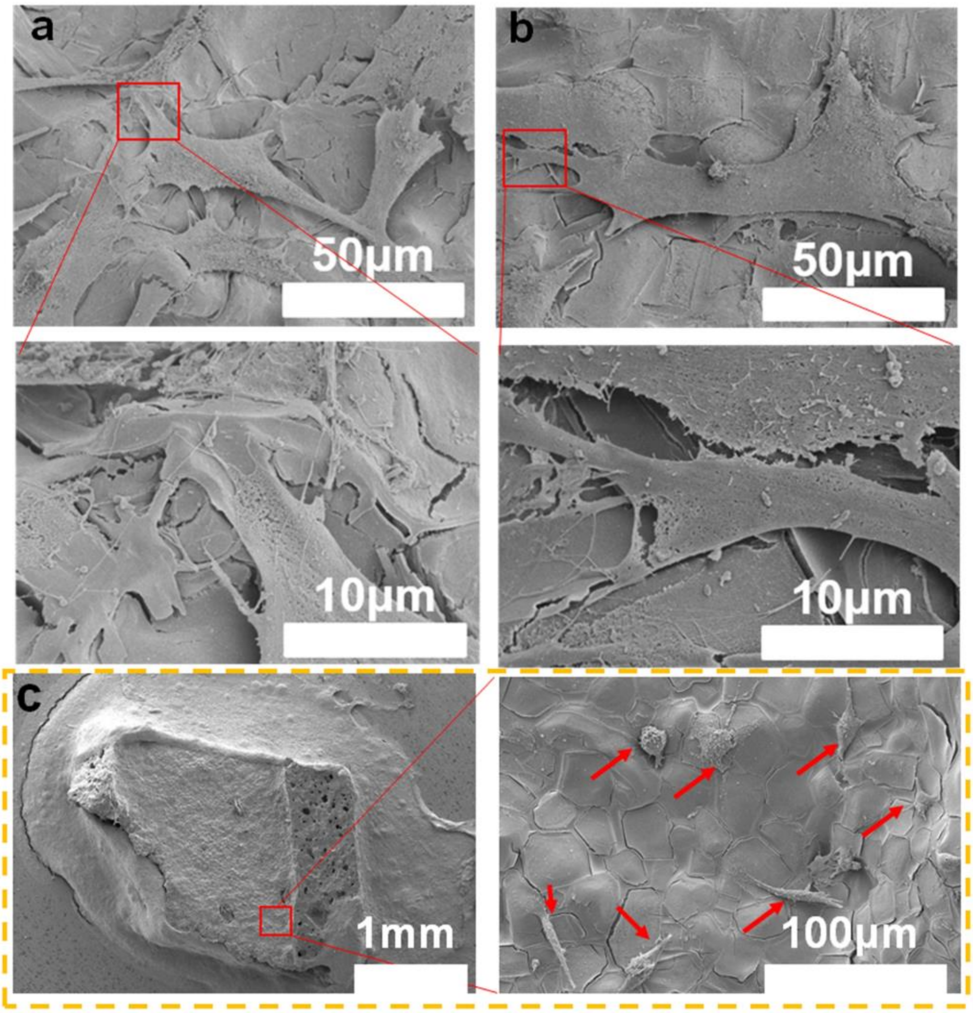

Figure S5. The rBMSCs adhesion on the outside of hollow tubes of HD-AKT (a) and HD-AKT/Ica (b). The cell spreading in the inside of tube wall of HD-AKT/Ica (c), in which the hollow tubes were broken in order to observe whether the cells can migrate in even the existence of bioceramic rods in the scaffolds. The results show that the existence of bioceramic rods in HD-AKT/Ica scaffolds will not block the cell migration.
